# 506 Methamphetamine Intoxication and Inpatient Outcomes for Burn Patients

**DOI:** 10.1093/jbcr/irae036.141

**Published:** 2024-04-17

**Authors:** Eloise Stanton, Yvonne L Karanas, Justin Gillenwater, Tam N Pham, Clifford C Sheckter

**Affiliations:** Keck School of Medicine of USC, Los Angeles, CA; Santa Clara Valley Medical Center, San Jose, CA; Keck Medicine of USC, Los Angeles, CA; University of Washington, Harborview Burn Centre, Seattle, WA; Stanford/Santa Clara Valley Medical Center, San Jose, CA; Keck School of Medicine of USC, Los Angeles, CA; Santa Clara Valley Medical Center, San Jose, CA; Keck Medicine of USC, Los Angeles, CA; University of Washington, Harborview Burn Centre, Seattle, WA; Stanford/Santa Clara Valley Medical Center, San Jose, CA; Keck School of Medicine of USC, Los Angeles, CA; Santa Clara Valley Medical Center, San Jose, CA; Keck Medicine of USC, Los Angeles, CA; University of Washington, Harborview Burn Centre, Seattle, WA; Stanford/Santa Clara Valley Medical Center, San Jose, CA; Keck School of Medicine of USC, Los Angeles, CA; Santa Clara Valley Medical Center, San Jose, CA; Keck Medicine of USC, Los Angeles, CA; University of Washington, Harborview Burn Centre, Seattle, WA; Stanford/Santa Clara Valley Medical Center, San Jose, CA; Keck School of Medicine of USC, Los Angeles, CA; Santa Clara Valley Medical Center, San Jose, CA; Keck Medicine of USC, Los Angeles, CA; University of Washington, Harborview Burn Centre, Seattle, WA; Stanford/Santa Clara Valley Medical Center, San Jose, CA

## Abstract

**Introduction:**

Methamphetamine intoxication frequently complicates inpatient burn admissions. While single-institution studies describe adverse outcomes during resuscitation, less is known about the associated inpatient complications and perioperative management in users. This analysis aims to identify these trends on a national level.

**Methods:**

The US National Trauma Data Bank was queried to identify burn patients between 2017-2021. Methamphetamine intoxication was identified on admission and compared to those who screen negative. Primary outcomes included death, stroke, and myocardial infarction (MI). Secondary outcomes included organ failure, timing of surgery, admission vitals, length of stay (LOS), and days in the intensive care unit (ICU). Multivariable regressions modeled the effects on outcomes adjusting for available covariates including demographics, total body surface area (TBSA) burned, and inhalation injury. Bonferroni adjustments were applied.

**Results:**

326,614 burn encounters were identified, and 5,380 (1.7%) had positive methamphetamine drug screens upon admission. Users were significantly older (35.2 versus 31.9 years, p < .001), had a greater percentage of males (85.3 vs. 82.0, p < .001), were more likely to have inhalation injury and overall had larger %TBSA burns (16% vs. 13%, (p < .001). Users had significantly higher but clinically insignificant ED systolic blood pressures (132.1 vs. 129.9, p < .001). There was no significant difference between the two groups regarding pulse rate >120 (1.7% vs. 1.6%, p=.611). Users were not more likely to die, sustain an MI, or stroke during admission. In contrast, users were significantly more likely to have alcohol withdrawal (0.8% vs. 0.5%, p=.019), drug withdrawal (0.3% vs. 0.2%, p=.003), a pulmonary embolism (PE) (0.9% vs. 0.7%, p=.039), and delirium (1.2% vs. 0.4%, p=.003). Users had longer times to surgery adjusting for burn severity and demographics (1.6 vs. 1.4 days, p=.033). There were no significant differences in the average number of wound coverage procedures (1.50 vs. 1.45, p=.947). Users had significantly longer hospitalization (8.7 vs. 6.2 days, p < .001); however, there was no significant difference between days in the ICU (6.7 vs. 6.4 days, p=.072).

**Conclusions:**

While not associated with mortality, methamphetamine intoxication was associated with an increased risk of many complications including PE, drug withdrawal, and delirium. Methamphetamine use was associated with delays in surgery and hospitalization.

**Applicability of Research to Practice:**

Recognize the increased risk of complications and the need for tailored perioperative care for methamphetamine users. Additionally, burn centers may consider how to avoid delaying care for users and support interventions for withdrawal.

